# Biological activity of 3-(2-benzoxazol-5-yl)alanine derivatives

**DOI:** 10.1007/s00726-021-03030-7

**Published:** 2021-07-08

**Authors:** Katarzyna Guzow, Ewa Mulkiewicz, Michał Obuchowski, Wiesław Wiczk

**Affiliations:** 1 Faculty of Chemistry, University of Gdańsk, Wita Stwosza 63, Gdańsk, 80-308 Poland; 2 Laboratory of Molecular Bacteriology, Intercollegiate Faculty of Biotechnology, Medical University of Gdańsk, Dębinki 1, Gdańsk, 80-211 Poland

**Keywords:** Benzoxazole, Amino acid, Antibiotic, Antifungal agent, Cytotoxicity

## Abstract

Searching for new drugs is still a challenge for science, mainly because of civilization development and globalization which promote the rapid spread of diseases, which is particularly dangerous in the case of infectious ones. Moreover, readily available already known antibiotics are often overused or misused, possibly contributing to the increase in the number of multidrug-resistant microorganisms. A consequence of this is the need for new structures of potential drugs. One of them is a benzoxazole moiety, a basic skeleton of a group of fluorescent heterocyclic compounds already widely used in chemistry, industry, and medicine, which is also present in naturally occurring biologically active compounds. Moreover, synthetic benzoxazoles are also biologically active. Considering all of that, a large group of non-proteinogenic amino acids based on 3-(2-benzoxazol-5-yl)alanine skeleton was studied in search for new antimicrobial and anticancer agents. Screening tests revealed that antibacterial potential of 41 compounds studied is not very high; however, they are selective acting only against Gram-positive bacteria (*B. subtilis*). Moreover, almost half of the studied compounds have antifungal properties, also against pathogens (*C. albicans*). Most of studied compounds are toxic to both normal and cancer cells. However, in a few cases, toxicity to normal cells is much lower than for cancer cells indicating these compounds as future anticancer agents. The research carried out on such a large group of compounds allowed to establish a structure–activity relationship which enables to select candidates for further modifications, necessary to improve their biological activity and obtain a new lead structure with potential for therapeutic use.

## Introduction

Civilization development and globalization significantly contribute to the rapid spread of diseases, especially infectious ones. Moreover, the readily available already known antibiotics are often overused or misused, resulting in an increase in the number of multidrug-resistant microorganisms. Therefore, the search for new effective drugs is still an important challenge for scientists.

**Fig. 1:**
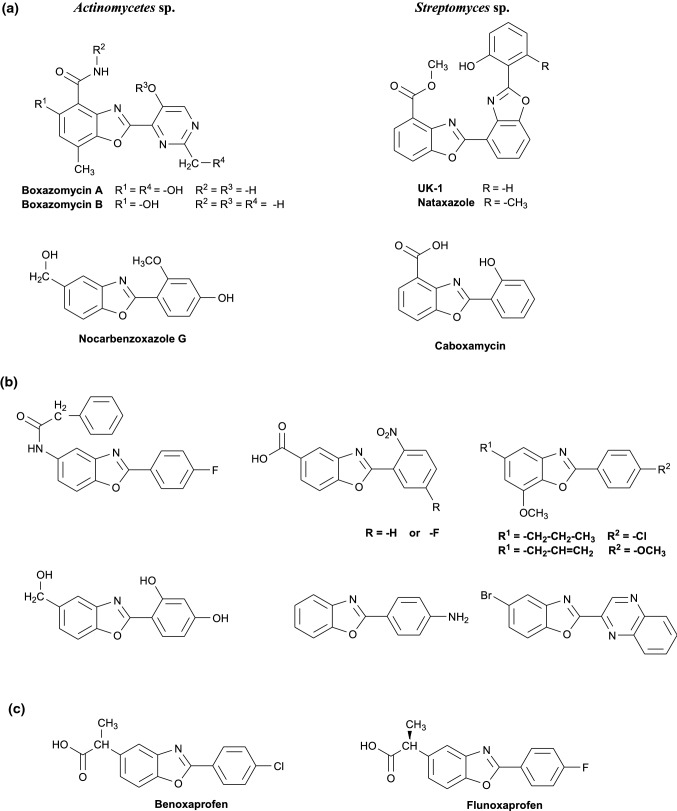
Structures of selected biologically active compounds containing benzoxazole moiety: **a** natural; **b** synthetic; **c** non-steroidal anti-inflammatory drugs

Among new structures of potential drugs, a benzoxazole moiety is interesting and promising one. This heterocyclic compound is a structural motif present in naturally occurring biologically active compounds isolated from marine sponges (Daletos et al. 2014; Pal et al. 2018; Takahashi et al. 2011) or gorgonian corals (Pal et al. 2018) as well as from bacteria (mainly *Actinomycetes* and *Streptomyces* species (Fig. 1a)—Chu et al. 2011; Hohmann et al. 2009; Pal et al. 2018; Sommer et al. 2008; Sun et al. 2015). Also, metabolites of some *Streptomyces* sp. (Sato et al. 2001; Ueki et al. 1993, 1997; Ueki and Taniguchi 1997) and antibiotics of calcimycin class (Prudhomme et al. 1984, 1986a, b; Sarma et al. 2003) contain benzoxazole ring. These compounds as well as their derivatives and analogues have mainly antibacterial, antifungal, antituberculotic, and/or anticancer activity (Pal et al. 2018; Prudhomme et al. 1984, 1986a, b; Reynolds et al. 1999; Sato et al. 2001; Sarma et al. 2003; Ueki et al. 1993, 1997; Ueki and Taniguchi 1997). Based on that, benzoxazole ring becomes a main skeleton of many synthetic derivatives (Fig. 1b) including some antibacterial and antifungal agents, in some cases even more active than widely used antibiotics (Arpaci et al. 2002a, b; de Carvalho et al. 2017; Jauhari et al. 2008; Kakkar et al. 2018; Kakkar and Narasimhan 2019; Kuroyanagi et al. 2010, 2011; Rida et al. 2005; Sattar et al. 2020; Şener et al. 2000; Tekiner-Gulbas et al. 2007; Temiz et al. 1998, 2008; Zhang et al. 2018). It was observed that some compounds based on benzoxazole have antituberculotic (Ertan-Bolelli et al. 2016; Pytela and Klimešová 2011; Rana et al. 2014a, b; Šlachtová and Brulíková 2018; Zhang et al. 2018), or antimalarial (Ongarora et al. 2015; Zhang et al. 2018), or antiviral activity (Bernard et al. 2014; Demmer and Bunch 2015; Jonckers et al. 2012; Sattar et al. 2020; Zhang et al. 2018). Moreover, it was demonstrated that a large group of benzoxazole derivatives exert cytotoxic effect on some cancer cells such as breast cancer cells (MCF-7, MDA-MB-213, MDA-MB-231), lung cancer cells (A549, H1975, HCC827), liver cancer cells (HepG2), prostate cancer cells (PC3), colorectal cancer cells (HCT-116, HT-29), oral cancer cells (KB), melanoma cells (A375), and others (Bernard et al. 2014; Chung et al. 2015; Desai et al. 2020; Giordano et al. 2019; Han et al. 2012; Kakkar et al. 2018; Kakkar and Narasimhan 2019; Kumar et al. 2002; Li et al. 2015; Omar et al. 2020; Reddy et al. 2016; Reynolds et al. 1999; Rida et al. 2005; Sachweh et al. 2015; Sato et al. 2001; Sattar et al. 2020; Sever et al. 2021; Slotkin et al. 2016; Ueki et al. 1993, 1997; Zhang et al. 2018; Zhong et al. 2020; Zi et al. 2019).

Among benzoxazole derivatives, there are potential anticonvulsant (Ibrahim et al. 2015; Sattar et al. 2020; Song et al. 2019; Zhang et al. 2018) or antipsychotic agents (Huang et al. 2015). They are also known as muscle-relaxant, sedative, diastolic, or anti-inflammatory agents (Dunwell et al. 1975a, b; Kaur et al. 2018; Sattar et al. 2020; Seth et al. 2014; Zhang et al. 2018). Moreover, benzoxazole moiety was a core pharmacophoric unit of commercially available non-steroidal anti-inflammatory drugs — benoxaprofen and flunoxaprofen (Fig. 1c, Dunwell et al. 1975a, b). However, because of the observed side effects of benoxaprofen (Lewis et al. 1990), both pharmaceuticals were withdrawn from the market. Nowadays, this heterocyclic ring is present in compounds tested as potential drugs for neurodegenerative diseases such as Alzheimer’s (Gutti et al. 2019; Zhang et al. 2018) or prion diseases (Hyeon et al. 2020).

Synthetic benzoxazole derivatives have often wide spectrum of antimicrobial activity. A large group of benzoxazole derivatives show antibacterial activity against Gram-positive bacteria (*Staphylococcus aureus*, *Streptococcus faecalis*, *Bacillus subtilis*) and/or Gram-negative bacteria (*Escherichia coli*, *Klebsiella pneumoniae*, *Pseudomonas aeruginosa*), and/or antifungal activity (*Candida albicans*, *Candida krusei*, *Candida glabrata*, *Candida-6*, *Candida-51*, *Aspergillus niger*, *Aspergillus flavus*), which in some cases is higher than the activity of the commercially available antimicrobial drugs used as controls. In many cases, a structure–activity relationship is observed and it was found that the most important are substituents in positions 2 and 5 of the benzoxazole ring, especially if both of them are occupied (Arpaci et al. 2002a; Demmer and Bunch 2015; Kuroyanagi et al. 2011; Sattar et al. 2020; Tekiner-Gulbas et al. 2007).

**Fig. 2:**
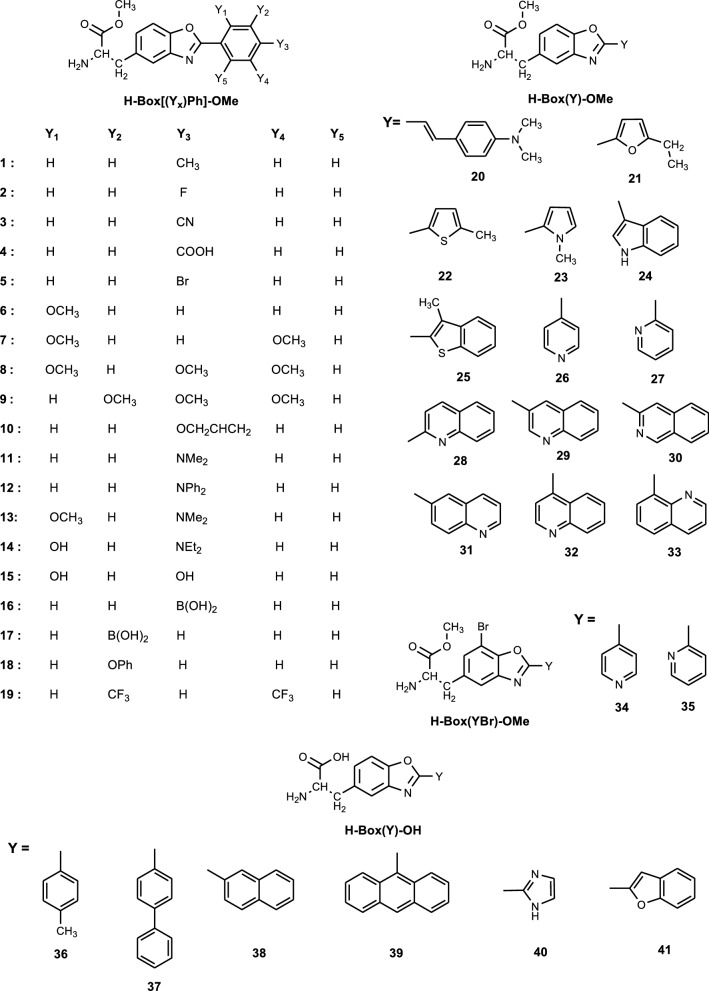
Structures of studied compounds

Taking these facts into account, we decided to study biological activity of a large group of non-proteinogenic amino acid derivatives based on benzoxazole skeleton. All 41 derivatives of 3-(2-benzoxazol-5-yl)alanine contain in position 5 of the benzoxazole amino acid moiety, whereas in position 2, various substituents such as phenyl, hydrocarbon, or heterocyclic group (Fig. 2).

## Materials and methods

### Synthesis

All studied compounds (Fig. 2) were obtained previously (Guzow et al. 2001, 2002, 2003, 2004, 2005a, b, 2007, 2012). To improve their solubility in water, the protecting groups were removed. For all compounds, the protection of α-amino group (Boc) was removed selectively by acidic hydrolysis using trifluoroacetic acid in dichloromethane (50:50 (v/v)) (Guzow et al. 2003, 2012). Additionally, in a few cases (compounds **36**–**41**, Fig. 2), first, the protection of α-carboxyl group (methyl ester) was removed using basic hydrolysis (1 M NaOH in methanol) (Guzow et al. 2003). The purity of the obtained compounds was at least 96%. It was checked by means of RP-HPLC (Varian) with UV detection (223 nm) using analytical Kromasil column (C-8, 5 µm, 250 mm long, i.d. = 4.5 mm). The mobile phase was a gradient running from 0.1% water solution of trifluoroacetic acid (phase A) to 80% of acetonitrile in phase A (phase B) over 60 min. The identification of all products was based on mass spectra recorded on Bruker Biflex III (MALDI-TOF) or MASSLAB TRIO-3 (FAB) instrument. In each case, (M + H)^+^ or M^+^ ion was detected.

### Microbiological studies

Antibacterial and antifungal assays were performed in vitro. First, the screening tests using *Bacillus subtilis* ATCC 23857 and *Escherichia coli* ATCC 25922 as model Gram-positive and Gram-negative bacteria, respectively, and yeast *Pichia pastoris* as model fungus were performed for all compounds. Such strains were chosen as representatives of the two largest groups of bacteria (*B. subtilis* and *E. coli*) and primitive *Eucaryota* (*P. pastoris*). Then, the active compounds were selected and tested against most common pathogens such as *Staphylococcus aureus* ATCC 25923, *Enterococcus faecalis* ATCC 29212, *Pseudomonas aeruginosa* ATCC 27853, and *Candida albicans* ATCC 10231.

Bacterial strains were subcultured overnight prior to the assay in Mueller–Hinton broth (Difco) at 37 °C. *Pichia pastoris* and *Candida albicans* were subcultured overnight at 25 °C in Mueller–Hinton and Luria–Bertani (Difco) broth, respectively, supplemented with 2% of glucose.

Screening tests against model microorganisms as well as against pathogens were carried out on the appropriate broth solidified by agar using antibiogram method and twofold serial dilution technique (Wiegand et al. 2008). Water solution of each studied compound (5 μl) was deposited on absorbent paper disk (Whatman 3MM) which was placed on Petri dish with medium inoculated with appropriate microorganism (50 μl of inoculum prepared by diluting the subcultured microorganism in its incubation broth to optical density at 570 nm (OD_570_) equal to 1). Each dish contains six paper disks — maximal concentration of the compound and its five serial dilutions. After the incubation for 24 h at 37 °C (bacteria) or 48 h at 25 °C (fungi), diameters of growth inhibition zones were measured.

The active compounds were selected and their minimal inhibitory concentration (MIC) values were determined in 96-well flat-bottomed microtiter plates using twofold serial dilution technique. Each compound was dissolved in appropriate broth, then serially diluted and 50 μl of each solution was transferred in duplicate to the wells. The microbial inoculum (OD_570_≈0.01) was added to the samples to achieve a final volume of 100 μl. Every test plate contains growth (inoculated broth) and blank (only studied compound in broth) controls. After 24 h incubation at 37 °C (bacteria) or 25 °C (fungi), optical density was measured at 570 nm using Elx800 Absorbance Reader (Bio-Tek Instruments). The lowest concentration of studied compound that completely inhibited growth of microorganism in well was taken as MIC value.

### Cytotoxicological studies

Cytotoxicity was determined using four different mammalian cell lines: rat glioma (C6), mouse fibroblasts (A9), human embryonic kidney (Hek293), and human dermal fibroblasts (HDF). The cells were grown as a monolayer in low-glucose (HDF) or high-glucose (C6, A9, Hek293) DMEM medium supplemented with 1% antibiotic solution (penicillin/streptomycin), 1% glutamine, and 10% fetal bovine serum (FBS) at 37 °C in a humidified atmosphere of 5% CO_2_. All culture media and supplements were obtained from Gibco. The medium was changed every 2 days and cells were subcultured. For the cytotoxicity assays, cells were seeded in 96-well plates at an initial density of 2.5 × 10^4^ (C6, A9) or 4 × 10^4^ (HDF, Hek293) cells per ml of appropriate culture medium and incubated for 24 h.

A colorimetric assay with WST-1 reagent (2-(4-iodophenyl)-3-(4-nitrophenyl)-5-(2,4-disulfophenyl)-2H-tetrazolium monosodium salt, Roche Diagnostics) was used for the cell viability tests. Stock solutions of the tested compounds were prepared in growth media with 0.5% DMSO added to improve solubility. Cells were exposed to nine different concentrations (from 2 μM to 10 mM) of the tested compounds. Each incubation, including blank and growth controls, was performed in triplicate. The cells were incubated for 44 h. After this time, 10 μl of WST-1 reagent, was added to each well and incubated for 4 h at 37 °C. Subsequently, the absorbance at 450 nm was measured in the plate reader. Cell viability was calculated as the percentage of the viability of exposed cells *vs.* controls. These data are the means of three independent experiments conducted for each compound. Concentration response curves were fitted with the nonlinear least squares method using a logistic model for the relation of cell viability and inhibition to the decadic logarithm of the tested concentrations (Ranke et al. 2004). The log EC_50_ values (EC_50_—half maximal effective concentration) were given, since it is a model parameter in the logistic model. Calculations were carried out with R language and environment for statistical computing (http://www.r-project.org).

## Results and discussion

### Antimicrobial activity

Antimicrobial activity of all studied 3-(2-benzoxazol-5-yl)alanine derivatives was screened using model bacterial strains, *Bacillus subtilis* (Gram-positive) and *Escherichia coli* (Gram-negative), as well as yeast *Pichia pastoris* (representative of *Eucaryota*). It was found that only a few compounds were active. Minimal inhibitory concentrations (MIC) obtained for them are presented in Table 1.

**Table 1:** Antimicrobial activity (MIC) of studied compounds against selected microorganisms

Compound		*Bacillus subtilis*		*Pichia pastoris*		*Candida albicans*	
		MIC		MIC		MIC	
No.	Designation	(μg/ml)	(μM)	(μg/ml)	(μM)	(μg/ml)	(μM)
**2**	H-Box[(4-F)Ph]-OMe	na	na	63	200	na	na
**6**	H-Box[(2-OMe)Ph]-OMe	na	na	146	448	na	na
**7**	H-Box[2,5-(OMe)_2_Ph]-OMe	na	na	150	421	na	na
**8**	H-Box[2,4,5-(OMe)_3_Ph]-OMe	6000	15544	na	na	na	na
**9**	H-Box[3,4,5-(OMe)_3_Ph]-OMe	na	na	190	492	na	na
**13**	H-Box[(2-OMe-4-NMe_2_)Ph]-OMe	190	515	46	125	196	531
**21**	H-Box(Fur)-OMe	na	na	125	398	na	na
**23**	H-Box(Pyrrol)-OMe	na	na	113	378	na	na
**26**	H-Box(4Py)-OMe	na	na	160	539	na	na
**28**	H-Box(2Q)-OMe	1100	3170	110	317	200	576
**29**	H-Box(3Q)-OMe	na	na	2370*	6830*	na	na
**30**	H-Box(3iQ)-OMe	na	na	140	403	na	na
**31**	H-Box(6Q)-OMe	na	na	3950*	11383*	na	na
**32**	H-Box(4Q)-OMe	na	na	1270*	3660*	na	na
**33**	H-Box(8Q)-OMe	1050	3026	400	1153	na	na
**34**	H-Box(4PyBr)-OMe	na	na	65	173	138	367
**35**	H-Box(2PyBr)-OMe	na	na	352	936	na	na

*Estimated value — concentration determined basing on tests on solidified broth because of too low solubility of the compound in the liquid broth

Performed tests revealed that antibacterial potential of these compounds is rather low as only four derivatives were active (H-Box[2,4,5-(OMe)_3_Ph]-OMe (**8**), H-Box[(2-OMe-4-NMe_2_)Ph]-OMe (**13**), H-Box(2Q)-OMe (**28**), H-Box(8Q)-OMe (**33**)) and their MIC values were very high (except for H-Box[(2-OMe-4-NMe_2_)Ph]-OMe (**13**), Table 1). However, it was observed that they are selective being active only against *B. subtilis* (Gram-positive bacteria). The activity of these four compounds against pathogenic strains (*S. aureus*, *E. faecalis*, *P. aeruginosa*) was also studied, but the results were negative.

Antifungal properties of studied compounds were much more pronounced in comparison with antibacterial ones. Among 41 studied compounds, 16 showed activity against *P. pastoris* with MIC values much lower than these determined for *B. subtilis* (Table 1). Moreover, the structure–activity relationship was observed (Table 1). Among 20 derivatives with substituted phenyl in position 2 of the benzoxazole (compounds **1**–**20**, Fig. 2), only 5 were active (compounds **2**, **6**, **7**, **9**, **13**, Fig. 2, Table 1). Four of these compounds (**6**, **7**, **9**, **13**) have electron-donating substituents (methoxy, dimethylamino group), whereas only one (compound **2**) has electron-accepting substituent (fluor). The most active compound was H-Box[(2-OMe-4-NMe_2_)Ph]-OMe (**13**) which also exhibited the highest antibacterial activity. Lack of dimethylamino group in position 4 of the phenyl ring of the tested compound, as in the case of H-Box[(2-OMe)Ph]-OMe (compound **6**, Fig. 2), significantly decreases the activity of the compound (about threefold, Table 1). The presence of additional methoxy group in the phenyl ring (H-Box[2,5-(OMe)_2_Ph]-OMe (compound **7**, Fig. 2)) does not affect much the antifungal activity of the compound as opposed to change in their positions, which has a greater effect (H-Box[3,4,5-(OMe)_3_Ph]-OMe (compound **9**) — active, H-Box[2,4,5-(OMe)_3_Ph]-OMe (compound **8**) — not active, Table 1). The slight decrease in MIC value (about 30%) observed for compound **9** with 3 electron-donating methoxy groups may be due to the steric hindrance. Among compounds with hydrocarbon in position 2 of the benzoxazole ring (compounds **36**–**39**, Fig. 2), no one was active which may be a result of steric hindrance of such substituent and/or lack of methyl ester at the carboxyl group.

In the case of derivatives containing heterocyclic substituent in position 2 (compounds **21**–**35** and **40**–**41**, Fig. 2), the activity was observed mainly for these with azaaromatic group (compounds **23**–**35**, Table 1). The presence of a substituent with sulfur (compound **22**, Fig. 2) or two heteroatoms (compound **40**, Fig. 2) makes the compound inactive (Table 1), similarly as in the case of a substituent made of two condensed rings of different sizes (5 and 6-membered, compounds **24**, **25** and **41**, Fig. 2). Compounds with 5-membered heteroaromatic substituent have similar activity regardless of the heteroatom (oxygen (**21**) or nitrogen (**23**), Table 1). In the case of azaaromatic derivatives, replacing 5-membered ring (compound **23**, Fig. 2) with 6-membered one (compound **26**, Fig. 2) causes a significant decrease in the compound activity (Table 1). However, introducing bromine atom (electron-accepting substituent) in position 7 of the benzoxazole ring results in the activity increase giving the most active compound (H-Box(4PyBr)-OMe (**34**)) among heterocyclic derivatives of benzoxazolylalanine (Table 1). Minimal inhibitory concentration value for H-Box(4PyBr)-OMe (compound **34**) was almost three times lower compared to H-Box(4Py)-OMe (compound **26**), whereas H-Box(2Py)-OMe (compound **27**) did not show any activity in contrast to H-Box(2PyBr)-OMe (compound **35**, Table 1). It suggests that antimicrobial activity depends on the position of the nitrogen atom in the substituent. This should be also true for quinolinyl derivatives; however, due to the poor solubility of 3 compounds in the medium used MIC determination and clear analysis of the results was difficult. To estimate the influence of the nitrogen atom position on the activity of the compound, the amount of each quinolinyl derivative present on the paper disk during the screening tests inhibiting yeast’s growth was calculated. The results are as follows: H-Box(2Q)-OMe (compound **28)**—11 μg, H-Box(3Q)-OMe (compound **29**)—12 μg, H-Box(3iQ)-OMe (compound **30**)—24 μg, H-Box(6Q)-OMe (compound **31**)—20 μg, H-Box(4Q)-OMe (compound **32)**—6 μg, H-Box(8Q)-OMe (compound **33**)—64 μg. Their analysis showed that more active are compounds with nitrogen atom present in the ring directly connected to the benzoxazole moiety (4Q, 2Q, 3Q, 3iQ, Fig. 2).

Only 3 out of 17 tested compounds, namely H-Box[(2-OMe-4-NMe_2_)Ph]-OMe (compound **13**), H-Box(2Q)-OMe (compound **28**), H-Box(4PyBr)-OMe (compound **34**), exhibited activity against pathogenic yeast *C. albicans*. Moreover, their antifungal activity (reflected in MIC values) was about two or four times lower comparing to their effect observed for *P. pastoris* (Table 1).

### Cytotoxicological studies

To assess whether studied benzoxazolylalanine derivatives are potential anticancer agents, the cytotoxic effect of all studied compounds, except four derivatives with very low solubility, was screened using rat glioma cell line (C6). It was found that 7 compounds were not cytotoxic in the studied concentration range (H-Box[2,4-(OH)_2_Ph]-OMe (**15**), H-Box[(4-Me)Ph]-OH (**36**), H-Box(BiPh)-OH (**37**), and H-Box(2-Bfur)-OH (**41**) (2–500 μM), H-Box(2-Naph)-OH (**38**, 4–1000 μM), H-Box[(4-COOH)Ph]-OMe (**4**, 20–5000 μM), and H-Box(2-Im)-OH (**40**, 40–10000 μM) (Table 2). Among them are mainly compounds without C-terminal methyl ester suggesting that the state of α-carboxyl group is important for such activity. It is also confirmed by the results obtained for the compounds **1** (H-Box[(4-Me)Ph]-OMe – cytotoxic) and **36** (H-Box[(4-Me)Ph]-OH – not cytotoxic).

**Table 2:** Cytotoxicity (EC_50_) of studied compounds to selected cell lines

Compound		EC_50_ [μM]			
No.	Designation	Rat glioma (C6)	Mouse fibroblasts (A9)	Human dermal fibroblasts (HDF)	Human embryonic kidney (Hek293)
1	H-Box[(4-Me)Ph]-OMe	309 ± 7	nd	nd	nd
2	H-Box[(4-F)Ph]-OMe	372 ± 3	nd	nd	nd
3	H-Box[(4-CN)Ph]-OMe	286 ± 7	nd	nd	nd
4	H-Box[(4-COOH)Ph]-OMe	No effect	nd	nd	nd
5	H-Box[(4-Br)Ph]-OMe	230 ± 1	80 ± 5	46 ± 0.3	238 ± 15
6	H-Box[(2-OMe)Ph]-OMe	438 ± 20	334 ± 22	100 ± 2	718 ± 92
7	H-Box[2,5-(OMe)_2_Ph]-OMe	900 ± 21	354 ± 33	60 ± 4	256 ± 21
8	H-Box[2,4,5-(OMe)_3_Ph]-OMe	832 ± 58	1700 ± 25	238 ± 9	745 ± 135
9	H-Box[3,4,5-(OMe)_3_Ph]-OMe	317 ± 36	1459 ± 19	81 ± 5	878 ± 70
10	H-Box[(4-OAll)Ph]-OMe	185 ± 4	nd	nd	nd
11	H-Box[(4-NMe_2_)Ph]-OMe	277 ± 6	nd	nd	nd
12	H-Box[(4-NPh_2_)Ph]-OMe	–^a^	nd	nd	nd
13	H-Box[(2-OMe-4-NMe_2_)Ph]-OMe	239 ± 11	97 ± 3	29 ± 1	314 ± 15
14	H-Box[(2-OH-4-NEt_2_)Ph]-OMe	337 ± 8	nd	nd	nd
15	H-Box[2,4-(OH)_2_Ph]-OMe	No effect	nd	nd	nd
16	H-Box[(4-B(OH)_2_)Ph]-OMe	299 ± 8	627 ± 26	101 ± 18	Not toxic^b^
17	H-Box[(3-B(OH)_2_)Ph]-OMe	59 ± 3	62 ± 7	88 ± 15	672 ± 58
18	H-Box[(3-OPh)Ph]-OMe	No effect	nd	nd	nd
19	H-Box[3,5-(CF_3_)_2_Ph]-OMe	–^a^	nd	nd	nd
20	H-Box(4-NMe_2_Cin)-OMe	229 ± 5	nd	nd	nd
21	H-Box(Fur)-OMe	450 ± 10	nd	nd	nd
22	H-Box(Tio)-OMe	954 ± 44	nd	nd	nd
23	H-Box(Pyrrol)-OMe	418 ± 48	278 ± 32	110 ± 2	852 ± 80
24	H-Box(Indol)-OMe	–^a^	nd	nd	nd
25	H-Box(Benzotio)-OMe	79 ± 7	53 ± 1	6 ± 0.4	437 ± 32
26	H-Box(4Py)-OMe	1866 ± 86	1174 ± 21	112 ± 9	4099 ± 131
27	H-Box(2Py)-OMe	983 ± 2	662 ± 49	296 ± 9	1429 ± 104
28	H-Box(2Q)-OMe	131 ± 5	50 ± 3	37 ± 0.1	326 ± 19
29	H-Box(3Q)-OMe	299 ± 24	51 ± 3	21 ± 1	378 ± 18
30	H-Box(3iQ)-OMe	147 ± 3	58 ± 4	54 ± 3	290 ± 10
31	H-Box(6Q)-OMe	–^a^	nd	nd	nd
32	H-Box(4Q)-OMe	567 ± 20	106 ± 10	26 ± 2	583 ± 29
33	H-Box(8Q)-OMe	377 ± 22	313 ± 22	394 ± 9	198 ± 5
34	H-Box(4PyBr)-OMe	1760 ± 8	408 ± 3	73 ± 12	3216 ± 171
35	H-Box(2PyBr)-OMe	965 ± 44	234 ± 21	48 ± 4	1980 ± 112
36	H-Box[(4-Me)Ph]-OH	No effect	nd	nd	nd
37	H-Box(BiPh)-OH	No effect	nd	nd	nd
38	H-Box(2-Naph)-OH	No effect	nd	nd	nd
39	H-Box(9-Ant)-OH	320 ± 15	nd	nd	nd
40	H-Box(2-Im)-OH	No effect	nd	nd	nd
41	H-Box(2-Benzofur)-OH	No effect	nd	nd	nd

*nd* not determined

^a^Too low solubility in the conditions of the experiment

^b^In the concentration range of 10–2500 μM

For cytotoxic compounds, the structure–activity relationship was observed (Table 2). Among phenyl derivatives of benzoxazolylalanine (compounds **1**–**20**, Fig. 2), these with phenyl substituted only in position 4 (compounds **1**–**3**, **5**, **10**–**12**, **16,** and **20**) have similar cytotoxicity, in most cases regardless of the type of the substituent (Table 2). Introducing additional substituents to the phenyl ring results in diminishing cytotoxicity, except for substitution in position 3 which seems to be the most important one (compounds **8**–**9** and **13**–**15**). Such substituted compounds are more cytotoxic as in the case of H-Box[3-B(OH)_2_Ph]-OMe (compound **17**) which cytotoxic effect is five times higher than this of H-Box[4-B(OH)_2_Ph]-OMe (compound **16**, Table 2). Toxicity of derivatives with double substituted phenyl depends on both the position and the type of substituents (compounds **7**, **13**–**15** and **19**, Fig. 2, Table 2). In such cases, the most important seems to be the substitution of position 4 of the phenyl as the presence of additional group in position 2 of the phenyl (H-Box[(2-OMe-4-NMe_2_)Ph]-OMe (**13**)) slightly increases cytotoxicity of the compound, whereas lack of its dimethylamino group in position 4 (H-Box[(2-OMe)Ph]-OMe (**6**)) causes twofold reduction of compound’s cytotoxicity (Table 2). Also, changing these substituents for hydroxyl and diethylamino groups (H-Box[(2-OH-4-NEt_2_)Ph]-OMe (**14**)) or two hydroxyl groups (H-Box[2,4-(OH)_2_Ph]-OMe (**15**)) causes a significant decrease in cytotoxicity (Table 2).

Benzoxazolylalanine derivatives containing in position 2 small heterocyclic substituent (furan, thiophene, pyrrole) are relatively not very cytotoxic (compounds **21**–**23**, Table 2). The least toxic among them is H-Box(Tio)-OMe (**22**); however, the exchange of thiophene for benzothiophene (compound **25**) makes the compound the most toxic one in this group. In the case of derivatives with oxygen as a heteroatom in the substituent (compounds **21** and **41**), an inverse relationship is observed (Table 2).

Cytotoxicity of derivatives with azaaromatic substituents depends on a size of the heterocyclic ring (compounds **23**, **24** and **26**–**35**). Compound with five-membered ring as a substituent (H-Box(Pyrrol)-OMe (**23**)) is more toxic than these with six-membered ones (H-Box(2Py)-OMe (**27**) and H-Box(4Py)-OMe (**26**)). However, introducing azaaromatic substituent larger than pyridine (quinoline — compounds **28**–**33**) significantly increases cytotoxicity of the compound (Table 2). Cytotoxicity of both of these derivatives (pyridyl and quinolinyl) depends on the position of the nitrogen in the aromatic ring: the closer to the benzoxazole ring, the higher toxicity of the compound (Table 2). Also, introducing the bromine atom (electron-accepting substituent) in position 7 of the benzoxazole ring (compounds **34** and **35**) slightly increases the cytotoxicity of the pyridyl derivatives (Table 2).

Based on the results of the screening tests described above, 19 compounds were selected for further studies performed for three normal cell lines (A9, HDF, and Hek293). Cells of A9 line are more sensitive to the presence of majority of studied compounds than these of C6 line (Table 2). The exceptions are three phenyl derivatives (H-Box[2,4,5-(OMe)_3_Ph]-OMe (**8**), H-Box[3,4,5-(OMe)_3_Ph]-OMe (**9**), H-Box[4-B(OH)_2_Ph]-OMe (**16**)) which are less toxic for A9 cell line. However, the structure–activity relationships observed for A9 cell line are similar as these for C6. For phenyl derivatives, the importance of substituent in position 3 of the phenyl as well as the presence of the dimethylamino group in position 4 is more pronounced than in the case of A9 cell line (EC_50_ value for H-Box[3-B(OH)_2_Ph]-OMe (**17**) is ten times lower than EC_50_ value for H-Box[4-B(OH)_2_Ph]-OMe (**16**)). Also, more than one group of the same type in the phenyl ring causes a decrease in cytotoxicity (compounds **7** and **8**, Table 2). Furthermore, compounds with electron-donating substituents in the phenyl ring are less toxic than these with electron-accepting groups (Table 2). In the case of azaaromatic derivatives, the greater impact of the presence of the bromine atom in position 7 of the benzoxazole ring was observed for A9 cell line (almost 3 times lower EC_50_ value for pyridyl derivatives with the bromine atom, Table 2). Moreover, exchanging smaller to larger azaaromatic group (pyridine to quinoline) in position 2 of the benzoxazole ring causes significant cytotoxicity increase [compounds **26** and **32** (about 11 times), **27** and **28** (about 13 times), Table 2].

Influence of studied benzoxazolylalanine derivatives on normal cell lines was assessed to establish whether their application as antimicrobial or anticancer agents would be safe for humans. As both application to the skin and administration of the drug were taken into account, human dermal fibroblasts (HDF) and human embryonic kidney (Hek293) cell lines were selected for the study. It should be emphasized that understanding potential renal toxicity is particularly important as unmetabolized drugs are largely excreted in the urine. Among studied compounds, only one (H-Box-[4-B(OH)_2_Ph]-OMe (**16**)) was not toxic to Hek293 cell line (Table 2). This cell line was much less sensitive to the presence of studied compounds (except H-Box(8Q)-OMe (**33**)) than HDF cell line (Table 2). Also, the majority of studied derivatives were less cytotoxic to Hek293 than for cancer C6 as well as for normal A9 cell lines. The opposite relationship was observed for HDF cell line (Table 2). Moreover, the majority of studied derivatives has EC_50_ value lower than MIC value indicating on greater cytotoxicity of these compounds than their antimicrobial potential (Tables 1 and 2).

Toxicity to HDF and Hek293 cell lines depends on the structure of the compound. Among methoxyphenyl derivatives, these with double substituted phenyl ring are the most cytotoxic ones. As in the case of A9 cell line, phenyl derivatives with electron-accepting substituents are more cytotoxic than these with electron-accepting groups (Table 2). Similarly as for cancer cell line, H-Box-[3-B(OH)_2_Ph]-OMe (**17**) is more toxic than H-Box-[4-B(OH)_2_Ph]-OMe (**16**) for HDF and Hek293 cell lines (Table 2). Azaaromatic derivatives of benzoxazolylalanine (compounds **23**, **26**–**30** and **32**–**35**) are less toxic than compound with benzothiophene as a substituent (compound **25**, Table 2). Cytotoxicity of azaaromatic derivatives depends on the size of heterocyclic ring. Compounds with 5-membered ring (pyrrole, **23**) are more toxic than these with 6-membered ring (pyridine, **26** and **27**). However, increasing the size of the azaaromatic substituent (compounds **28**–**30**, **32** and **33**) results in greater cytotoxicity of such compound (quinolinyl derivatives, Table 2). Moreover, azaaromatic compounds in which substituent’s nitrogen atom is in spatial proximity to the benzoxazole ring (compounds **27**–**30** and **33**) are more toxic, except for toxicity of pyridyl derivatives to HDF cell line (Table 2). Introducing the bromine atom in position 7 of the benzoxazole ring (compounds **34** and **35**) causes significant increase of compound’s toxicity to HDF cell line. In the case of Hek293 cell line, this effect is less pronounced and observed only for H-Box(4PyBr)-OMe (**34**). For H-Box(2PyBr)-OMe (**35**), the opposite effect is observed (Table 2).

## Conclusion

Performed screening tests concerning a large group of 3-(2-benzoxazol-5-yl)alanine derivatives (41 compounds) enabled to characterize biological activity of each compound and determine the influence of substituent in position 2 of the benzoxazole ring on it.

It was observed that studied compounds have small antibacterial potential in contrast to antifungal one. Moreover, it has been shown that the activity of the compound depends on its structure. The widest spectrum of activity has H-Box[(2-OMe-4-NMe_2_)Ph]-OMe (compound **13**) and H-Box(2Q)-OMe (compound **28**) which are active against *B. subtilis*, *P. pastoris,* and *C. albicans*. Lower minimal inhibitory concentration values make the first compound more promising as a potential antimicrobial agent. The majority of active benzoxazolylalanine derivatives are selective. Only antifungal properties are characteristic for 12 compounds of which the most important is H-Box(4PyBr)-OMe (compound **34**) which is also active against pathogenic *C. albicans* (the lowest MIC value). One compound (H-Box[2,4,5-(OMe)_3_Ph]-OMe (**8**)) has only antibacterial properties, but its high MIC value excludes its potential use as an antibiotic.

Most of studied compounds are toxic to both cancer and normal cells. For each cell line, the structure–activity relationship was observed. Also, it was found that fibroblasts are more sensitive to the presence of benzoxazolylalanine derivatives than the other studied cell types. Moreover, almost all studied compounds are the most toxic to the human dermal fibroblasts (HDF) what exclude application of their potential pharmaceutical preparation to the skin. In the case of Hek293 cell line, almost all compounds (except compounds **7**, **8** and **33**) are less toxic for this cells than for cancer cells. Among them is H-Box[3-B(OH)_2_Ph]-OMe (compound **17**) which toxicity to cancer cells is tenfold higher than to Hek293 cells and could be a candidate for anticancer agent. Similar difference between toxicity to cancer and normal cells is observed for a few more compounds. Among them, the most important is H-Box(4PyBr)-OMe (compound **34**) for which MIC value for pathogenic *C. albicans* is lower than its cytotoxic concentrations (except for HDF cell line). Toxicity of all the other antifungal compounds to normal cells (expressed as EC_50_ value) is higher than their antimicrobial potency (expressed as MIC values) which makes them rather useless as potential chemotherapeutic agents.

The obtained results indicate that substituent in position 2 of the benzoxazole ring has a great influence on the antimicrobial and anticancer activity of 3-(2-benzoxazol-5-yl)alanine derivatives. Also, the state of the α-carboxyl group (blocked or free) may be important. The structure–activity relationship established basing on studies on such a large group of compounds enabled to select candidates for further modifications. Performing additional optimization of another positions will allow to improve biological activity of these compounds and obtain a lead structure with potential for therapeutic use.
